# Automated Quantification of Human Osteoclasts Using Object Detection

**DOI:** 10.3389/fcell.2022.941542

**Published:** 2022-07-05

**Authors:** Sampsa Kohtala, Tonje Marie Vikene Nedal, Carlo Kriesi, Siv Helen Moen, Qianli Ma, Kristin Sirnes Ødegaard, Therese Standal, Martin Steinert

**Affiliations:** ^1^ TrollLABS, Department of Mechanical and Industrial Engineering, Faculty of Engineering, Norwegian University of Science and Technology (NTNU), Trondheim, Norway; ^2^ Centre of Molecular Inflammation Research, Department of Clinical and Molecular Medicine, Faculty of Medicine and Health Sciences, Norwegian University of Science and Technology (NTNU), Trondheim, Norway; ^3^ Vitroscope AS, Trondheim, Norway; ^4^ Department of Mechanical and Industrial Engineering, Faculty of Engineering, Norwegian University of Science and Technology (NTNU), Trondheim, Norway; ^5^ Department of Hematology, St. Olavs University Hospital, Trondheim, Norway

**Keywords:** osteoclasts, object detection, machine learning, artificial intelligence, automatic image analysis

## Abstract

A balanced skeletal remodeling process is paramount to staying healthy. The remodeling process can be studied by analyzing osteoclasts differentiated *in vitro* from mononuclear cells isolated from peripheral blood or from buffy coats. Osteoclasts are highly specialized, multinucleated cells that break down bone tissue. Identifying and correctly quantifying osteoclasts in culture are usually done by trained personnel using light microscopy, which is time-consuming and susceptible to operator biases. Using machine learning with 307 different well images from seven human PBMC donors containing a total of 94,974 marked osteoclasts, we present an efficient and reliable method to quantify human osteoclasts from microscopic images. An open-source, deep learning-based object detection framework called Darknet (YOLOv4) was used to train and test several models to analyze the applicability and generalizability of the proposed method. The trained model achieved a mean average precision of 85.26% with a correlation coefficient of 0.99 with human annotators on an independent test set and counted on average 2.1% more osteoclasts per culture than the humans. Additionally, the trained models agreed more than two independent human annotators, supporting a more reliable and less biased approach to quantifying osteoclasts while saving time and resources. We invite interested researchers to test their datasets on our models to further strengthen and validate the results.

## 1 Introduction

In order to be healthy, the skeleton is constantly undergoing a balanced remodeling process where bone is removed by osteoclasts (OCs) before osteoblasts are recruited to build new bone (Charles and Aliprantis, 2014; Marino et al., 2014). Overactivation of osteoclasts may lead to unbalanced bone remodeling and excessive loss of bone. This is seen in diseases such as rheumatoid arthritis, multiple myeloma and cancers metastasizing to bone (Yahara et al., 2022).

Osteoclasts are highly specialized, multinucleated cells originating from cells of the monocyte-macrophage lineage. Two key cytokines, macrophage colony-stimulating factor (M-CSF) and receptor activator of NFkB ligand (RANKL), are essential for osteoclast formation. M-CSF is important for survival and proliferation of osteoclast precursors and leads to the expression of RANK (Marino et al., 2014). Signaling through RANK by RANKL is required for fusion of the osteoclast precursors and differentiation into mature osteoclasts (Marino et al., 2014; Pereira et al., 2018).

Human osteoclasts can be differentiated *in vitro* from mononuclear cells isolated from peripheral blood or from buffy coat (Marino et al., 2014). Typically, monocytes are isolated from peripheral blood mononuclear cells (PBMCs) by adhesion to plastic or by anti-CD14 coated beads. The cells are subsequently cultured in the presence of M-CSF and RANKL for about 14–16 days until multinucleated cells appear (Sørensen et al., 2007; Marino et al., 2014). Osteoclasts are defined as cells containing three or more nuclei and are tartrate-resistant acid phosphatase (TRAP) positive (Marino et al., 2014). TRAP is an acid phosphatase secreted by osteoclasts during bone resorption (Hayman, 2008). As the name indicates, TRAP is resistant to tartrate inhibition, which makes it distinguishable from other acid phosphatases, a property exploited during TRAP-staining (Burstone, 1959; Filgueira, 2004; Hayman, 2008). TRAP-staining is one of the most common methods to characterize osteoclasts *in vitro* cell cultures (Filgueira, 2004; Hayman, 2008; Marino et al., 2014).

While the definition of osteoclasts may be straightforward, identifying and correctly quantifying the number of osteoclasts in culture is a challenge. The number and size of osteoclasts are usually evaluated under a light microscope, which requires trained personnel. It is time-consuming and subjective. To avoid operator bias, each sample must be counted blind by at least two individuals. Osteoclast number and size may also be evaluated after imaging of the cultures. The nuclei in each cell can be difficult to see using the traditional TRAP-staining protocol, but this challenge can be overcome by adding nuclear stains such as Hoechst 33,342. Using combined fluorescence and transmitted light imaging systems, digital brightfield images and fluorescence images can be merged. Still, counting osteoclasts based on such images is difficult and the same challenges with time consumption and operator bias exist. Thus, there is a great need for a more unbiased, efficient, and reliable method to quantify osteoclasts in culture.

A few previous studies have attempted to quantify OCs from microscopic images of animal cells automatically. Cohen-Karlik et al. (2021) used machine learning (ML) to train an object detection model for measuring the number and area of OCs in cell cultures of mice. Their approach achieved a high correlation with trained human annotators for detecting subclasses of OCs with different numbers of nuclei. However, they do not mention the model performance (accuracy, precision, etc.). The “OC_Finder” system by Wang et al. (2021) also detects osteoclasts from mice. It uses an automated cell segmentation approach before applying deep learning to classify the cells as OCs or non-OCs. Their system achieved 98.1% accuracy and correlated well with a human examiner. Emmanuel et al. (2021) used an artificial intelligence-assisted method through proprietary software to identify and count OC from Wistar rats, with no significant difference in accuracy compared to manual methods while saving time. To our knowledge, OCs from humans have not been analyzed using ML. Our images also contain more variation than the other methods’ datasets. Therefore, we have developed a method to quantify human osteoclast from a variety of experiments using ML-based object detection.

Object detection combines computer vision and ML techniques to locate and classify objects in images. Using samples of images with labeled objects, we can train an object detection model to detect the objects in new images automatically. The model consists of several parts, where a convolutional neural network (backbone) can learn and extract features from images, which is connected to a predictor (head) for estimating object location (bounding box) and class probabilities. We use an open-source object detection (deep learning) framework called Darknet (Alexey et al., 2021). Darknet has achieved state-of-the-art results with its recent YOLOv4 model (Bochkovskiy et al., 2020) and is known for its training and prediction speed on a single graphics processing unit (GPU). Darknet and versions of the YOLO models have been used in multiple fields, including medicine and diagnosis (Elgendi et al., 2020; Yao et al., 2022), agriculture (Zheng et al., 2021), construction and industry (Nath and Behzadan, 2020; Kohtala and Steinert, 2021), and autonomous vehicles (Cai et al., 2021) to name a few.

We trained, validated, and tested multiple object detection models using osteoclasts generated from seven human PBMC donors, resulting in 307 different wells. Each well was marked using Fiji (Schindelin et al., 2012), with 94,974 OCs in total, and automatically converted to training and test datasets. The models are thoroughly evaluated using various training scenarios to analyze their performance compared to human annotators and to discuss their potential for replacing the labor-intensive process of manually counting OCs.

## 2 Methods

### 2.1 *In Vitro* Methods

#### 2.1.1 Differentiation of Osteoclasts From CD14^+^ Cells—Dataset I

**FIGURE 1:**
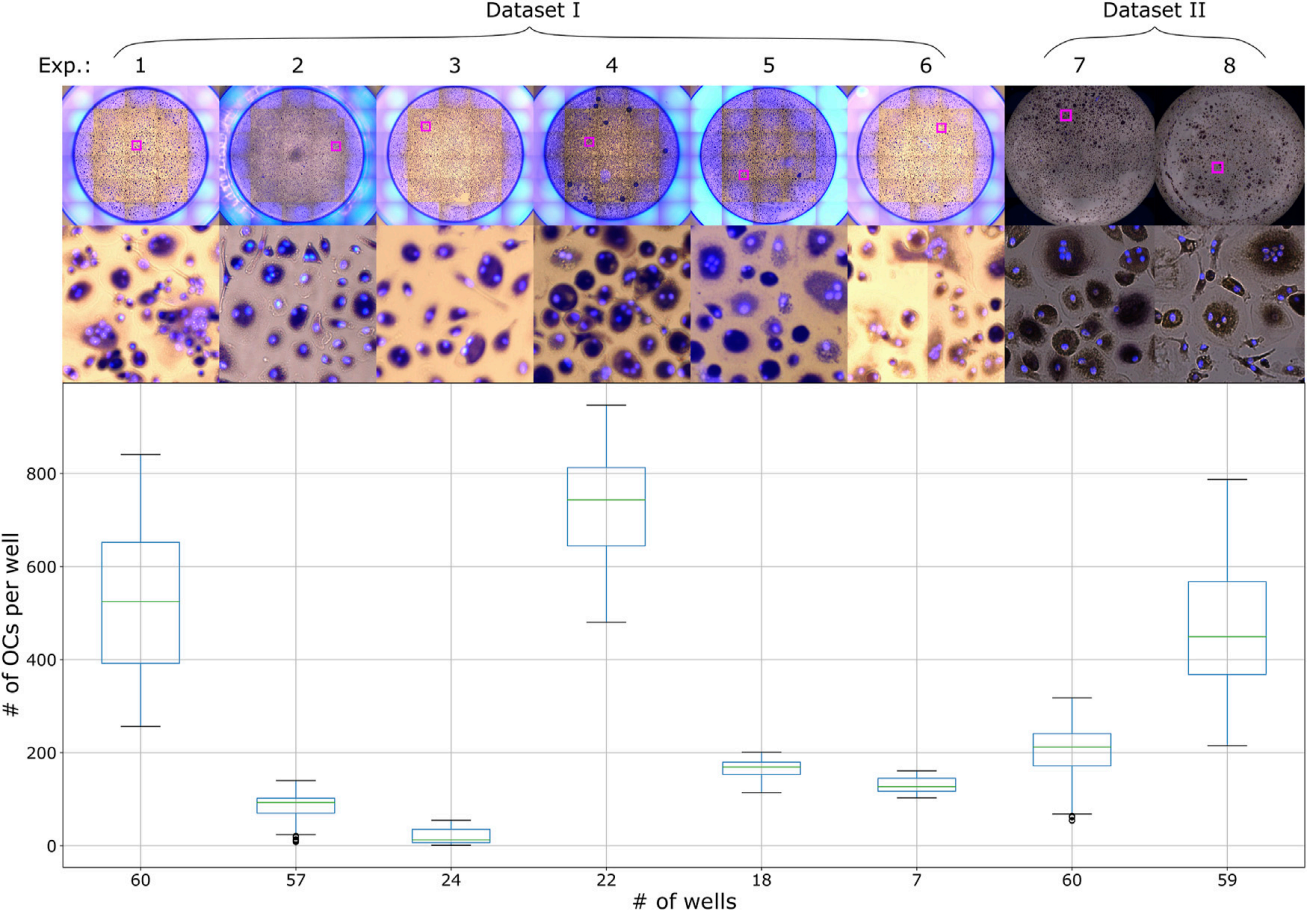
Distribution of the number of OCs per well with an example well image from each experiment in Dataset I and II.

Human buffy coats were provided by the Blood bank at St. Olavs Hospital (REK #2009/2245). PBMCs were isolated using a Lymphoprep density gradient (Alere), and CD14^+^ cells were isolated from these using CD14 microbeads (#130-050-201, Miltenyi Biotech). 13,000 CD14^+^ cells per well were seeded out in transparent plastic 96-well plates (#3599, Corning). The cells were cultured in α-minimum essential medium without phenol red (αMEM, #41061-029, Gibco) with 10% heat-inactivated pooled human serum supplemented with M-CSF (30 ng/ml, R&D Systems), RANKL (10 ng/ml, R&D Systems) and transforming growth factor- β (TGF β , 1 ng/ml, R&D Systems). The number of days used to differentiate the cells varied from donor to donor. Pre-osteoclasts were observed after 5–9 days. At this point, the cells were stimulated with various stimulants as part of an experiment. Mature osteoclasts were observed after about 12–16 days and the cells were TRAP stained. Six PBMC donors were used for dataset I, one per experiment presented in Figure 1.

#### 2.1.2 Differentiation of Osteoclasts From Macrophages—Dataset II

PBMCs were isolated as described above. The CD16^+^ patrolling monocytes (pMos) and CD16^−^CD14^+^ inflammatory monocytes (iMos) were isolated from one PBMC donor using CD16^+^ monocytes isolation kit (#130-091-765, Miltenyi Biotec) and CD14 microbeads in sequence. The purified cells were seeded out at 30,000 cells per well in black 96-well plates with glass bottom (#P96-1.5H-N, Cellvis). The cells were cultured in α-MEM medium with 10% heat-inactivated pooled human serum and M-CSF (10 ng/ml) for 7 days to form macrophages. At this point, the cells were stimulated with various stimulants as part of an experiment. Media was also changed to pre-osteoclastogenic differentiation medium containing M-CSF (30 ng/ml), RANKL (10 ng/ml) and TGF-β (1 ng/ml) before seven additional days of culture. The media supplements were then adjusted to M-CSF (10 ng/ml) and RANKL (50 ng/ml) and cells cultured three more days before TRAP staining. One PBMC donor was used for dataset II, the same donor for both experiments 7 and 8 presented in Figure 1. Experiment 7 represents pMos differentiated osteoclasts and experiment 8 represents iMos differentiated osteoclasts.

#### 2.1.3 Differentiation of Osteoclasts From Human Osteoclast Precursor Cells—Retraining Dataset

Human osteoclast precursor cells (#2T-110, Lonza) were plated at 10,000 cells per well in a transparent plastic 96-well plate and cultured according to the manufacturer’s instructions in OCP medium (#PT-8021, Lonza) in the presence of M-CSF (33 ng/ml) and RANKL (66 ng/ml). The cells were stimulated with various stimulants as part of an experiment during culture. When mature osteoclasts were present, the cells were TRAP stained. Two wells from this experiment were used for a small retraining of the model, described in Section 3.4.

#### 2.1.4 Staining

Mature osteoclasts were stained for TRAP using the Acid Phosphatase, Leukocyte (TRAP) Kit (#387A, Sigma-Aldrich) following the manufacturer’s instructions, with the following exceptions: osteoclasts were fixed with 4% paraformaldehyde (#43368, Alfa Aesar) in phosphate-buffered saline (PBS) for 15 min and appropriate TRAP staining was observed after incubation for up to 1.5 h. Cells were then washed twice with deionized H_2_O, before nuclei were stained with Hoechst 33,342 (#H3570, Life Technologies) 1:5,000 in PBS. Cells were kept in this solution during imaging.

#### 2.1.5 Imaging

Images were acquired with an EVOS FL Auto 2 Microscope (Invitrogen by Thermo Fisher Scientific) using a ×10 objective. TRAP staining was imaged using transmission microscopy, capturing a brightfield image of the well with a color camera. The EVOS light cube tagBFP was used for fluorescent detection of Hoechst. The microscope captured several smaller images of different regions of the well, which could later be arranged next to each other resulting in one larger image of the whole well, this process is called tiling. For dataset I and the Retraining dataset, tiling of the images was done in the EVOS software (Invitrogen EVOS FL Auto 2 Imaging System), creating two images per well, one TRAP image and one Hoechst image. For dataset II, tiling of the already merged images was done using a custom script in Fiji. TRAP and Hoechst images were merged using either the EVOS software or Fiji for all datasets, generating one image of the whole well. The edges of the wells were not imaged completely in dataset II, which resulted in these images being smaller than the images from dataset I. All images were saved as TIF files. The datasets I and II contain a total of 307 well images from eight different experiments, with their OC distribution and sample images shown in Figure 1. The Retraining dataset contain two wells from one experiment.

#### 2.1.6 Manual Osteoclast Counting

The merged images from EVOS were used for the human counting. Prior to counting, the images from an experiment were given random names using a Fiji script to make the counting unbiased. Fiji was used to count the osteoclasts by manually marking each osteoclast using the multi-point tool. When clicking on osteoclasts in an image, the multi-point tool leaves a mark and keeps track of the number of total markings in the image. When all wells of an experiment had been counted, the images could be decoded, revealing the results of the experiment.

### 2.2 Datasets and Training Procedures

#### 2.2.1 Data Preparation

**FIGURE 2:**
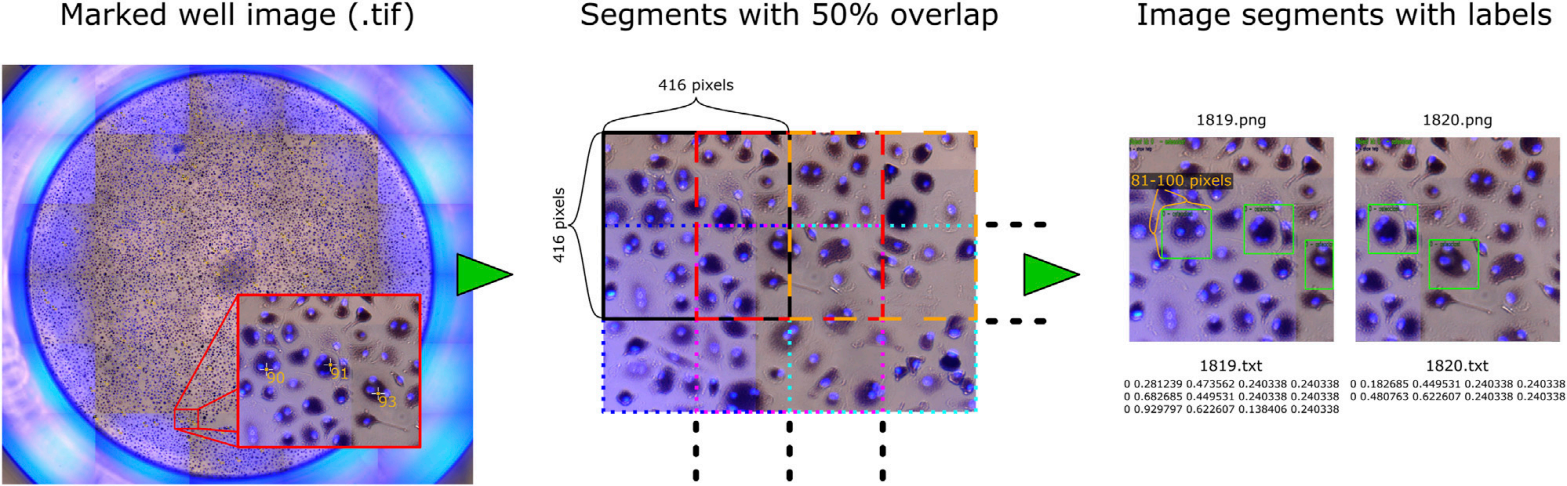
Sample image with processing steps, where each well image is split into overlapping segments that are saved as separate images, with each mark converted to a bounding box stored in a text file.

The two datasets (I and II) were initially intended for different research topics and purposes. This provided us the opportunity to test our approach on various OCs in a large number of images, and thus improve and thoroughly evaluate the algorithm. The well images were marked prior to this study, and their placements relative to the OCs are therefore not optimized for training an object detection model. We have not edited the marks due to the large number of samples (94,974 marked OCs) and the time required to correct each label manually. Therefore, several scripts were made to automatically create and prepare the data before training a model for detecting OCs. Each image of a well was processed, as illustrated in Figure 2.

First, a Fiji script converted the TIF images into PNG and exported every marked OC’s pixel coordinate to a CSV file. The PNG format removed the metadata (markings) and reduced file size for further processing. Because the object detection framework can only process small images, each image of a well was further split into segments of 416 × 416 pixels with 50% overlap, resulting in 2,200 segments for each image (10,248 × 9,122 pixels) in dataset I and 1,520 segments for each image (8,320 × 7,760 pixels) in dataset II. An overlap of 50% was used to ensure that each cell could be viewed entirely at least once while increasing the number and variation of training data. Object detection also requires a bounding box that covers the region of interest for detection. Based on observations, most cells would fit inside a box that is approximately 0.9756% of the width of the whole well image, which we used to automatically create bounding boxes covering roughly 100 pixels in height and width for dataset I and 81 pixels for dataset II. The bounding box coordinates around each marked OC were then saved to a text file corresponding to each image segment, described by its center, width, and height relative to the segment. Bounding boxes extending the perimeter of an image segment were reduced to fit within the segment. The two additional wells in the Retraining dataset with large variation in cell sizes were manually labeled with bounding boxes using LabelImg (Tzutalin, 2022) to ensure that each OC was covered entirely.

#### 2.2.2 Training Procedure

Transfer learning was used by training the OC detection models using an existing weights file for the convolutional layers [yolov4. conv.137 from Alexey et al. (2021)], which is pre-trained on the MS COCO (common objects in context) dataset (Lin et al., 2014). The pre-trained weights have learned to recognize many useful features from images to increase the training speed for detecting new objects. Training and prediction were performed locally on a laptop with an Intel i9-8950HK 2.9 GHz CPU connected to an external NVIDIA GeForce RTX 2080 Ti GPU.

We applied the same configuration file provided for the YOLOv4 model, with batch size 64, input width and height set to 416 pixels, and a learning rate of 0.001 for the first 80% training iterations, which is reduced by a factor of 10 for each remaining 10% of training iterations. Darknet also provides methods for using data augmentation during training, which will randomly alter the input images to increase the variability of the data and improve generalization. For data augmentation, we used random hue, saturation, exposure, cropping, aspect ratio, and mosaic data augmentation (randomly mixing four training images).

#### 2.2.3 Training Scenarios

Four separate training scenarios were analyzed. In the first scenario, a single model (M1) was trained and optimized for the entire dataset, where the well images from each experiment (see Figure 1) were split into a training (∼60%), validation (∼20%), and test (∼20%) set, with the total number of samples in the final datasets shown in Table 1. We trained the model for 11,073 iterations, i.e., two epochs when using a batch size of 64, and evaluated the validation set every 500 iterations for selecting the best model after training is complete. Finally, using the test set with M1, we can assess how closely the trained model can match the annotators (ground truth) for OC detection and counting. The 60/20/20 data split is the most common approach used to train (fit the model), validate (tune and select the best model), and test (unbiased measure of performance) ML models, and represents how the method would usually be applied in practice. Thus, our goal is to evaluate the applicability of using an open-source object detection framework to detect human OCs from microscopic images. By comparing the validation and test accuracies, we can also assess the model’s degree of bias (underfitting) and variance (overfitting).

**TABLE 1:** Number of samples used for each model.

Models	Train		Validation		Test		Sum	
	Wells	Segments	Wells	Segments	Wells	Segments	Wells	Segments
M1	183	354,320	64	124,480	60	115,680	307	594,480
M2_exp1, 2, 4	16	35,200	2	4,400	2	4,400	80	162,400
M2_exp8	16	24,320	2	3,040	2	3,040		
M3_P1, M3_P2	4	8,800	2	4,400	2	4,400	8	17,600

In many cases of ML, the available data does not represent the variation found in the wild (unseen samples), and it is uncertain how the chosen method generalizes to this new, unforeseen variation. However, with a large dataset from different experiments, it is possible to simulate this scenario by only training and validating the model on a subset of the available data and then testing it against “unseen” subsets. Thus, to assess the ability of our approach to generalize across different experiments (different donors and image appearance), we randomly picked 20 well images from experiments 1, 2, 4, and 8 for the second training scenario. A model was then trained (16 wells) and validated (2 wells) for each experiment (denoted by M2_exp1, M2_exp2, M2_exp4, and M2_exp8) and then tested against each other’s remaining two test wells. Each model was trained for 2500 iterations, i.e., 6.5 epochs for M2_exp8 and 4.5 epochs for the other models. This training scenario was performed to test how a model trained on only one experiment generalizes to other ‘unseen’ experiments or if the models tend to overfit if not introduced to more variation.

In the third scenario, we analyzed eight wells from experiment 4 that were marked independently by two annotators (P1 and P2). Two models were trained for each annotated dataset, where models M3_P1 and M3_P2 are trained on P1 and P2’s labels, respectively. We used 4-fold cross-validation (CV), using four training wells and two validation and test wells, to analyze each well without being part of the training and thus reduce bias. The models were trained for 3,000 iterations for each CV step (roughly 20 epochs). We can then directly compare both human-human and model-human agreements to assess if the proposed object detection method for automatic OC counting can replace humans and further evaluate the proposed method’s applicability and generalizability.

A fourth scenario was included to test if model M1 can be retrained to work on different-sized cells with larger variation. We labeled two wells from the Retraining dataset with large and small OCs containing at least three and up to more than 20 nuclei. The labels (bounding boxes used as ground truth) had to be made manually using a labeling tool since the auto-generated labels used for training the other models would not fit these cells. The cells ranged from 40 to 1,600 pixels in width and height, with an average of 260 pixels. The image segments were increased to 650 pixels in width and height to ensure that at least 95% of the labeled cells would fit inside. One of the wells was used to retrain and validate the model, while the other well was used to test the accuracy.

### 2.3 Evaluation

**FIGURE 3:**
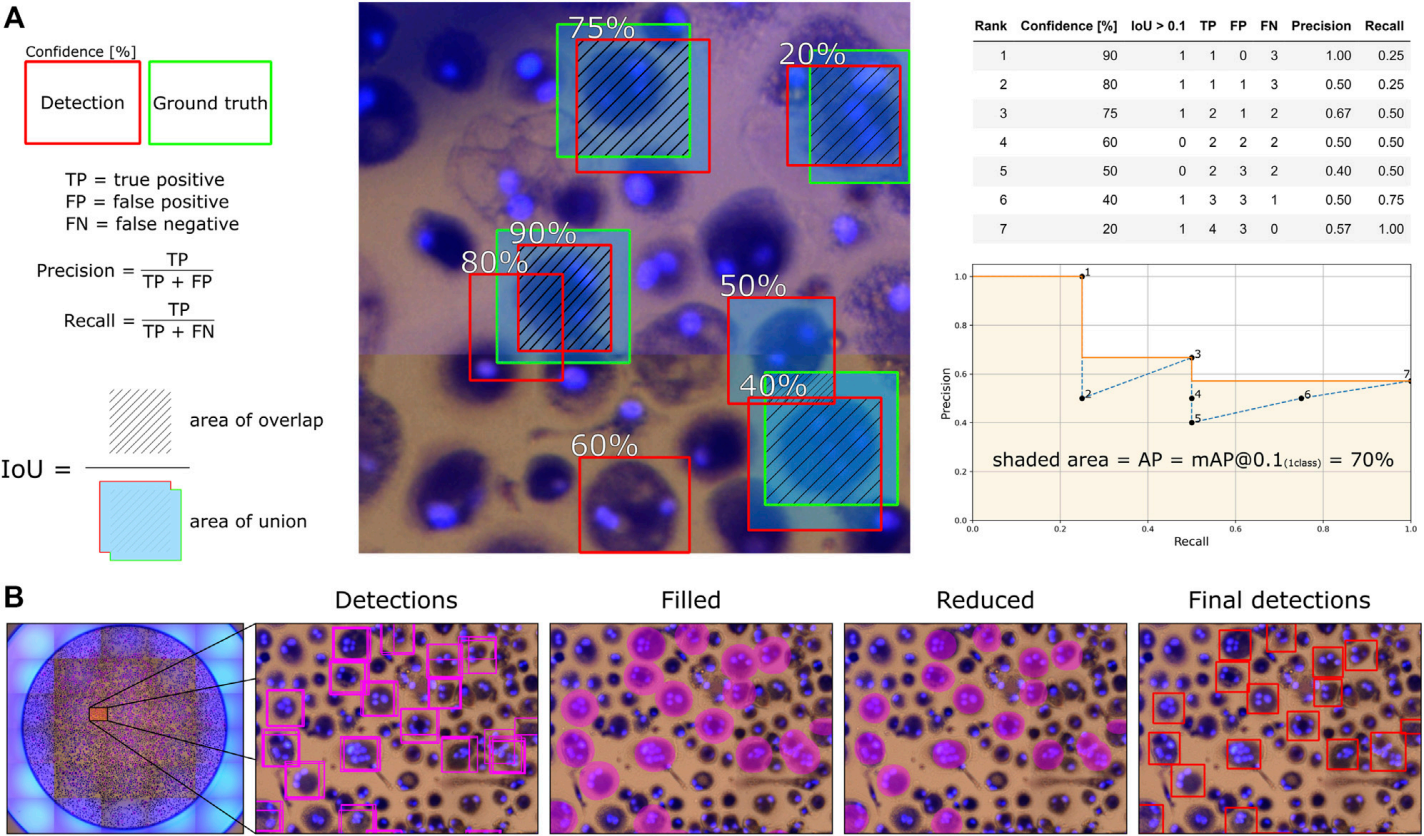
An illustrative example for how **(A)** average precision (mAP@0.1) is calculated and how **(B)** the total number of detected OCs are counted and segmented for each well with overlapping image segments.

Since the human-labeled marks from Fiji were not perfectly centered on the cells, and the bounding boxes were given a fixed size around the marks, we used a relatively low Intersection over Union (IoU) threshold of 0.1 for considering a detection a true positive. The mean average precision (mAP) is then calculated through Darknet, which employs the average precision (AP) calculation procedure provided by the PASCAL VOC2010 challenge (Everingham et al., 2015). Here, AP is the area under the monotonically decreasing precision-recall curve ranked by detection confidence. Subsequent detections of the same ground truth label are counted as false positives in the calculation. The mAP is the mean of the APs for all classes, and since we only consider one class, AP is equal to mAP. The mAP with an IoU threshold of 0.1 is referred to as mAP@0.1, which we calculate for the validation set during training to select the best model and then for the test set to report model performance. We also report mAP@0.5 results since an IoU threshold of 0.5 is commonly used for object detection models. An illustrative example of how mAP@0.1 is calculated for one sample image is shown in Figure 3A, where the same approach is used when multiple images are analyzed.

With the mAP metric considering every detection confidence and representing how accurately the trained model can locate, classify, and recall the marked OCs, we need to find a detection threshold for detecting OCs in new images. Therefore, we also calculate precision and recall values for the validation set to find the detection threshold that achieves the highest F1-score (harmonic mean of the precision and recall). We can then use this threshold value to detect and count the total number of OCs for each well in the test set by evaluating all corresponding image segments. Since the training images (segments) are overlapping, we compare the detection accuracy with and without overlapping segments to determine which approach is better suited. The number of segments in Table 1 is thus reduced by 75% in the case of only using non-overlapping segments. We created a simple algorithm using OpenCV (Bradski, 2000) to prevent multiple detections of the same OC when using overlapping segments. The algorithm first fills an ellipse-shaped region within the detected bounding boxes reduced by 20% in height and width to differentiate compact OCs with overlapping detections. The connected ellipses are then fused to reduce multiple detections of the same OC to one, finally creating bounding boxes around the merged ellipse shapes. The procedure is illustrated in Figure 3B.

After analyzing each whole well, we can compare the total number of OCs detected by the model and annotators and measure their agreement by calculating the root-mean-squared error (RMSE). *Python* (version 3.7.9) with the Scipy library (version 1.7.1) was used for calculating Pearson correlation coefficients between the models and annotators and perform normality tests.

## 3 Results

### 3.1 Scenario 1: Model Performance Using the Entire Datasets

**FIGURE 4:**
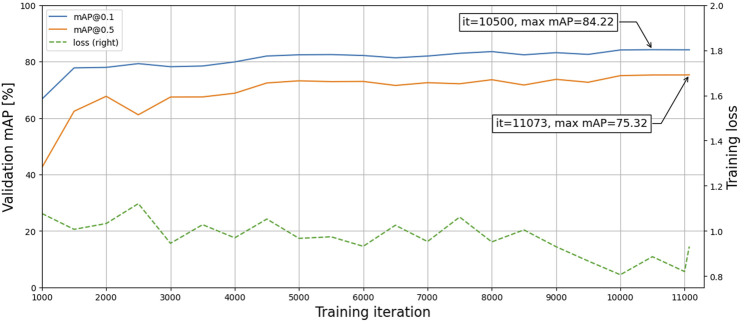
Training loss and validation accuracies during training.

The first model (M1) was trained for approximately 2 days and 14 h, including validation-set evaluations every 500 iterations taking roughly 2 h each time. Although the total training duration can be substantially reduced by excluding the validation set, selecting the best model based on training loss may cause overfitting. Since the validation set is not used to fit the model, it will reduce bias and better indicate model performance and generalization. Figure 4 shows the training loss and validation accuracy for the entire training duration. The best models were selected based on the highest validation accuracies and then evaluated on the test set, with results presented in Table 2. The mAP values were acquired on overlapping and non-overlapping validation and test images, with a mean absolute difference of only 0.2% between the approaches. Thus, the effect of the segmentation method does not significantly affect the mAP values. Furthermore, the similar and high validation and test accuracies indicate that the trained model has both low variance and bias, respectively, thus having generalized properly. The mAP@0.5 values are consistently lower than the mAP@0.1 values, which is expected due to the stricter evaluation criteria.

**TABLE 2:** Validation and test results for the best models.

IoU threshold	Validation					Test			
	mAP [%]	P	R	F1	DT	mAP [%]	P	R	F1
0.1	84.22	0.79	0.81	0.80	0.25	85.26	0.80	0.82	0.81
0.5	75.32	0.75	0.75	0.75	0.26	75.92	0.76	0.74	0.75

P, precision; R, recall; F1, F1 score; DT, detection threshold.

**FIGURE 5:**
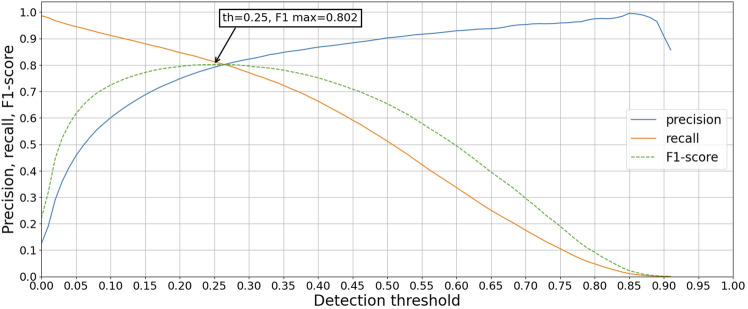
Precision, recall, and F1-score for the validation set using the model that achieved the highest mAP@0.1 score.

The precision, recall, and F1 scores were calculated for every detection threshold incremented by 0.05, with the results shown in Figure 5 for the validation set using the best model (highest mAP@0.1 score). The highest F1 score was achieved with a detection threshold of 0.25.

**FIGURE 6:**
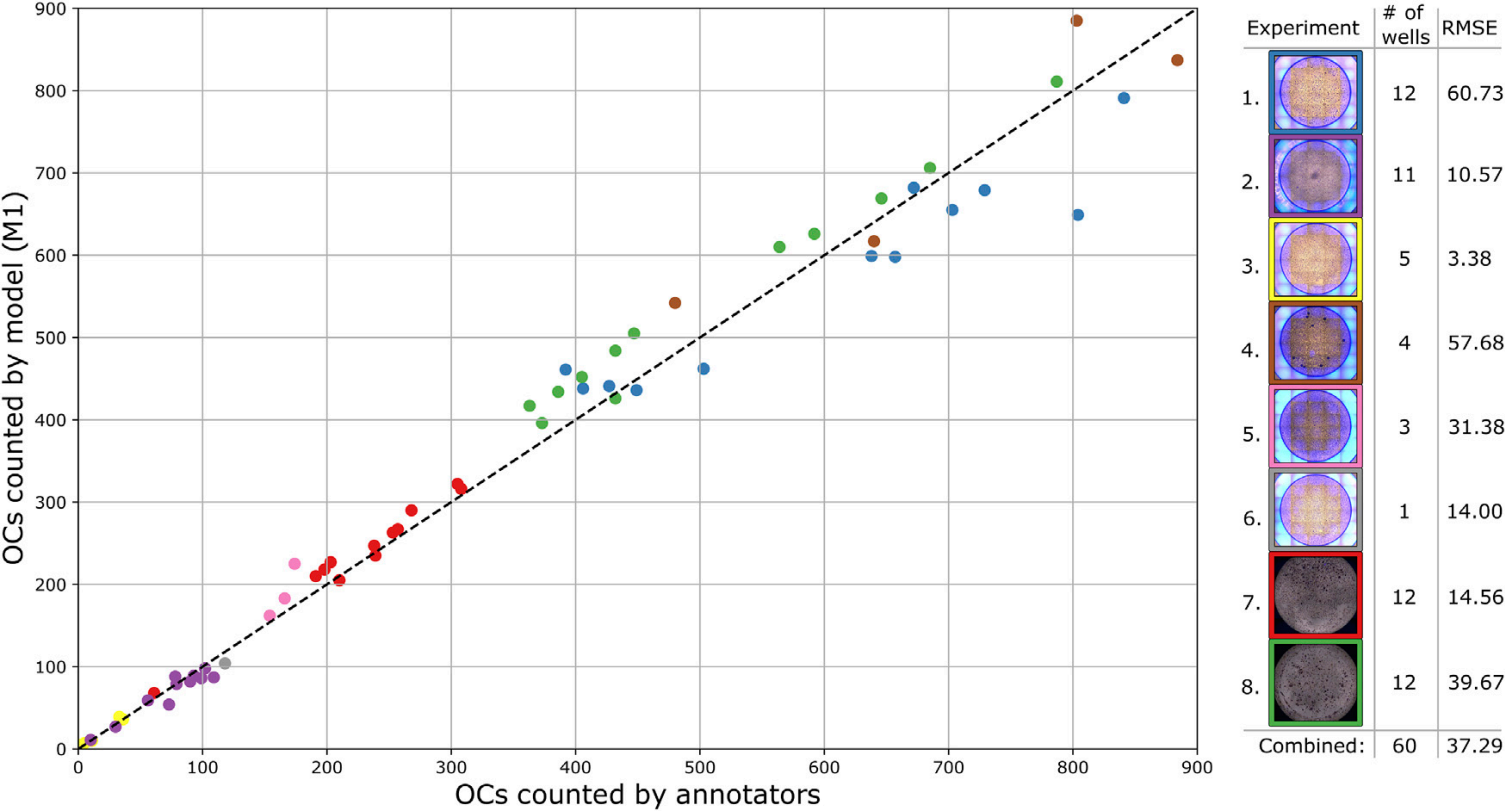
Comparison of the total number of detected OCs by the trained model and annotators for each test well, with each experiment separated by color. The dashed diagonal line represents the line of perfect agreement.

Using the best model based on mAP@0.1 and a detection threshold of 0.25, the number of OCs was detected and counted for each validation well. The process shown in Figure 3B, applied to overlapping segments, resulted in a combined RMSE of 51.87 while detecting directly on non-overlapping segments resulted in a combined RMSE of 32.37. Therefore, the approach of using overlapping segments for detection was disregarded, as it deviates more from the annotators than using non-overlapping segments without the extra step of combining detections. Figure 6 shows the number of OCs counted by the model compared to the annotators for each test well, with RMSE calculated for every experiment. The combined RMSE of all test wells is 37.29, with a Pearson correlation coefficient of 0.99 (*p* < 0.001, from scipy. stats.pearsonr) between M1 and annotators. These results support the applicability of using object detection to match human-level accuracy for counting OCs.

**FIGURE 7:**
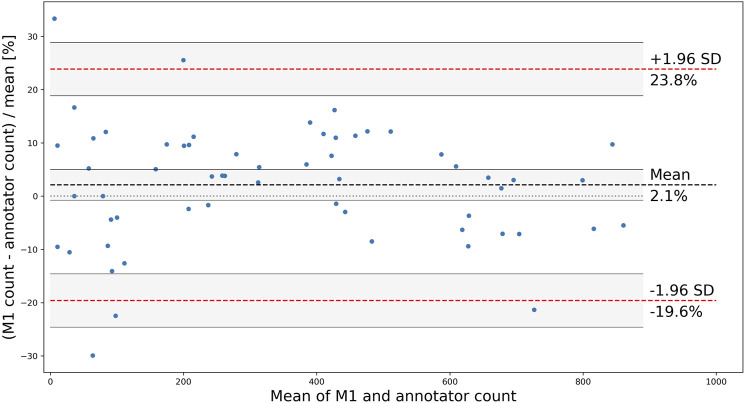
Bland and Altman plot showing the difference between M1 and annotators for each counted test well, with the limits of agreement shown as red, dashed lines, mean (bias) shown as a black dashed line, and CIs shaded in grey.

Although the correlation between the trained model and human annotators is high and positive, it does not necessarily imply a good agreement on the total number of counted OCs. To further analyze the applicability of the proposed method, a Bland and Altman plot is shown in Figure 7 with confidence interval (CI) calculations based on Giavarina (2015). The plot shows the difference (in the percentage of the mean) between measurements for the test wells, for which the residuals are normally distributed (*p* > 0.18, from scipy. stats.normaltest). The limits of agreement (mean difference ±1.96 SD) are 23.8% and −19.6%, which contain 95% of the differences. The bias of 2.1% is not significant since the line of equality (representing perfect agreement) is within its CI, indicating that the model consistently counts the number of OCs close to the human annotators without a systematic difference. However, the agreement interval is relatively wide, with the most considerable deviations found for the wells containing the fewest OCs. One outlier having a mean of three (one annotated and five detected OCs) and a difference of 133.3% was removed from the analysis.

### 3.2 Scenario 2: Generalization Across Experiments

**FIGURE 8:**
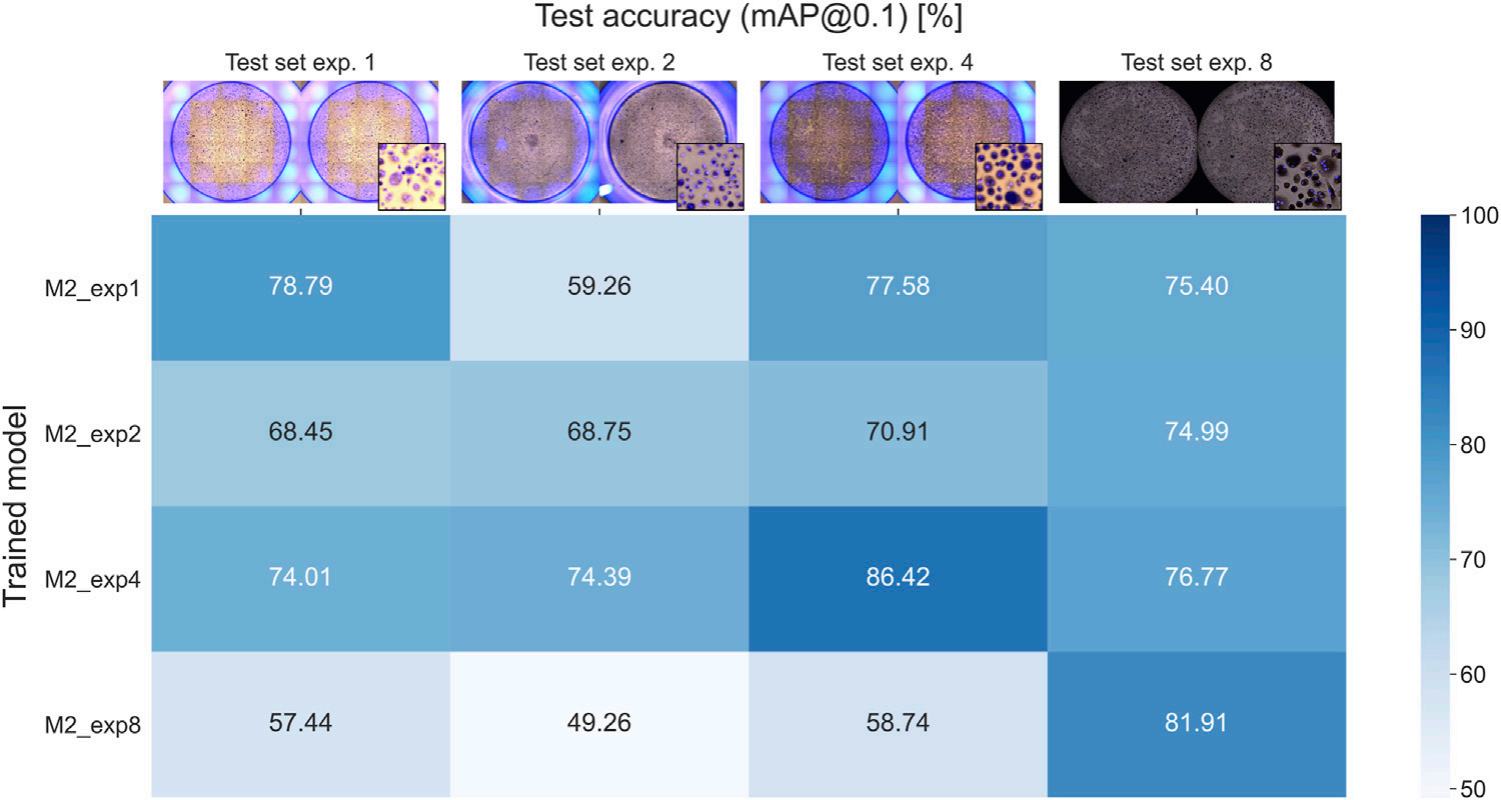
Test accuracies for models trained and validated on one experiment each.

Four models were trained, each using 16 samples from only one experiment (1, 2, 4, and 8 in Figure 1), and then evaluated against test wells from each experiment. The resulting mAP@0.1 values are shown in Figure 8. It is expected that a model trained, validated, and tested on the same experiment will achieve higher test accuracy compared to the other experiments since the training and test data are more related. However, this is not the case for model M2_exp2, which scored higher on experiments 4 and 8.

Additionally, each model achieves high accuracies on the test wells from experiment 8. In contrast, the model trained with images from experiment 8 has the lowest accuracies when tested on the other experiments. The dark appearance of the images in experiment 8 reduced the effectiveness of data augmentation (random hue, saturation, and exposure) when training M2_exp8, resulting in the model not being able to generalize properly. At the same time, the other experiments are not affected on the same level and were, therefore, able to generalize sufficiently to detect OCs from experiment 8 with reasonable accuracy. These results show that the method can produce models able to detect OCs from different experiments that were not part of the training and validation, thus showing generalizable tendencies of the trained models.

### 3.3 Scenario 3: Cross-Validation With Two Human Annotators

**FIGURE 9:**
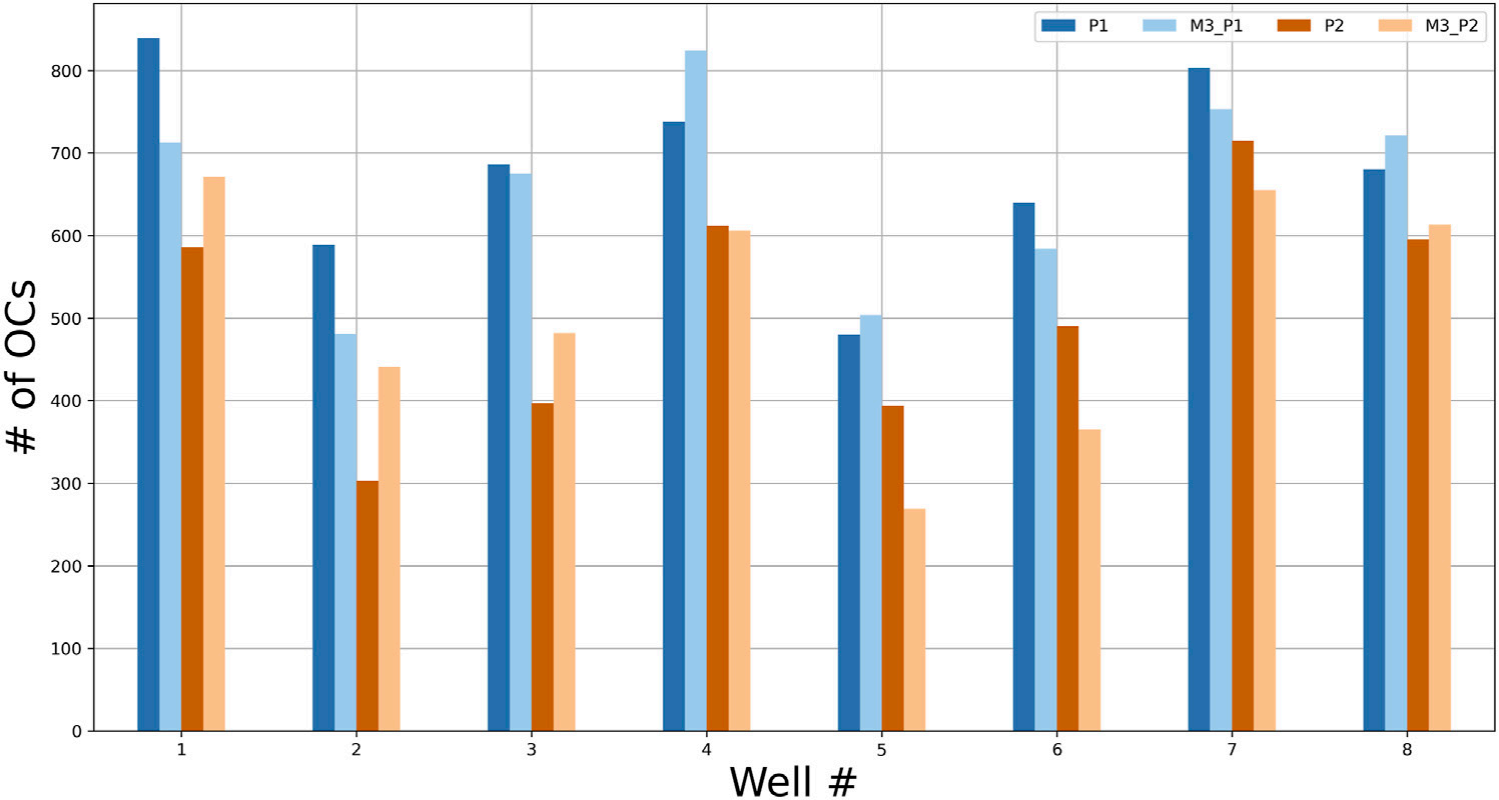
The number of OCs counted by two annotators (P1 and P2) and the two models M3_P1 and M3_P2 for each test well from experiment 4.

Two models were trained with a 4-fold CV using eight wells from experiment 4, marked independently by two annotators. The trained models, M3_P1 and M3_P2, had an average test accuracy (mAP@0.1) of 85.5 and 77.78%, respectively. The total numbers of OCs counted by the annotators and models are shown in Figure 9, with RMSE and correlation coefficients between each OC counter shown in Table 3. As seen in Table 3, the models agree more with the person marking the dataset, and the models agree more than the two annotators.

**TABLE 3:** Agreement between each OC counter, ranked by lowest RMSE.

	RMSE	Mean difference ±SD	Pearson correlation coefficient
P1 vs. M3_P1	73.27	25.00 ± 73.63	0.81
P2 vs. M3_P2	92.67	−1.25 ± 99.06	0.76
P2 vs. M3_P1	161.36	−145.38 ± 74.86	0.84
M3_P1 vs. M3_P2	163.01	144.13 ± 81.43	0.83
P1 vs. M3_P2	178.78	169.13 ± 61.97	0.92
P1 vs. P2	190.42	170.38 ± 90.90	0.76

### 3.4 Scenario 4: Retraining and Detection of Varying OC Sizes

**FIGURE 10:**
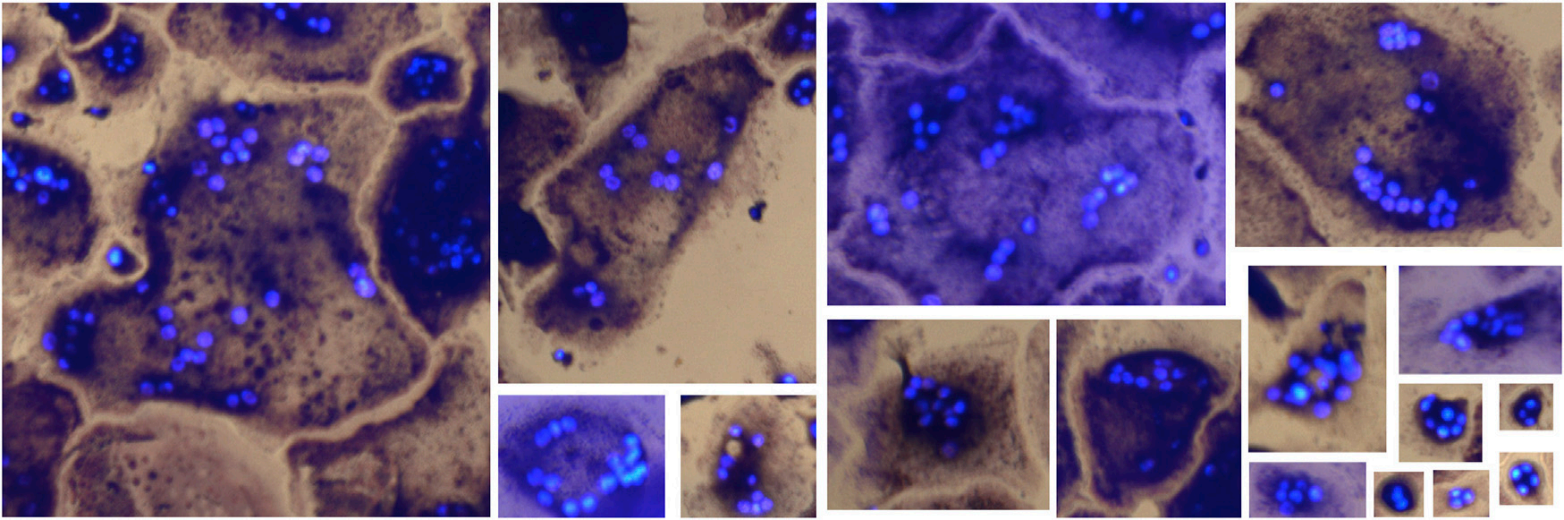
OCs of various sizes detected (true positives) by the retrained model.

Model M1 applied directly to the two wells of the Retraining dataset with large variation in osteoclast size resulted in 66.27% mAP@0.1, an F1-score of 0.66, and counted 16% fewer OCs than the annotator. M1 was then retrained using one of the wells for 50 min, where the highest validation accuracy (88.77%) was found at the 25 min mark. This resulted in a new test accuracy of 91.71% mAP@0.1, an F1-score of 0.85, and counted 11% more OCs than the annotator. These promising results support the applicability of the proposed method for detecting OCs with various shapes and sizes. A few sample detections from the test well are shown in Figure 10, with OCs ranging from 626 pixels to 64 pixels in width.

## 4 Discussion

### 4.1 Automatic OC Counting Accuracy

Our approach can automatically detect and count human OCs from microscopic images with good accuracy. The Pearson correlation coefficient of 0.99 indicates that the trained model can reliably count the number of OCs compared to human annotators, which is essential when comparing the amount of OCs between experiments. Furthermore, the trained model can locate and classify OCs in various images with a mAP@0.1 accuracy of 85.26% and count the number of OCs with a bias of only 2.1%. However, the limits of agreement found in Figure 7 ranges from −19.6 to 23.8%, and their acceptability must be based on the study being conducted. These results are auspicious when considering the suboptimal labels used for training, suggesting that the model can be improved further by optimizing the labels. The model can also be retrained to account for various cell sizes, as shown in scenario four. However, due to the few samples used, more manually labeled data is needed to further improve detection of various OC sizes.

One well image (550 segments without overlap) takes about 49 s to analyze, including 28s for splitting the image and saving each segment to a folder and 21s to perform detection (∼26 frames per second) and count the total number of OCs. Manual counting takes 15–40 min depending on the number of cells, whereas our approach takes around 6 min on the CPU. Even without GPU acceleration, our approach can save a lot of time and manual labor, especially when analyzing multiple wells.

By training several models using only one experiment each, we showed in scenario two that the method will still produce models able to detect OCs on different experiments and images with reasonable accuracy. Therefore, we expect that the model (M1) trained on all our experiments will work well on completely new experiments that may look slightly different. It is, however, essential to validate the model and tune the detection threshold on new images before relying on the reported accuracy. We also found that the darker images produced the least accurate models due to the reduced effect of data augmentation, which should be considered when capturing new data and training new models for cell analysis.

A trained model will naturally agree more with the person annotating the dataset, as seen in Figure 9 and Table 3. However, the trained models agreed more than the two annotators, indicating that the models have consistency at least as good as the human annotators and were able to learn the essential features for detecting OCs without overfitting on erroneous or missing labels. This further supports the use of trained object detection models to replace the manual process without losing accuracy.

### 4.2 Limitations and Typical Errors

**FIGURE 11:**
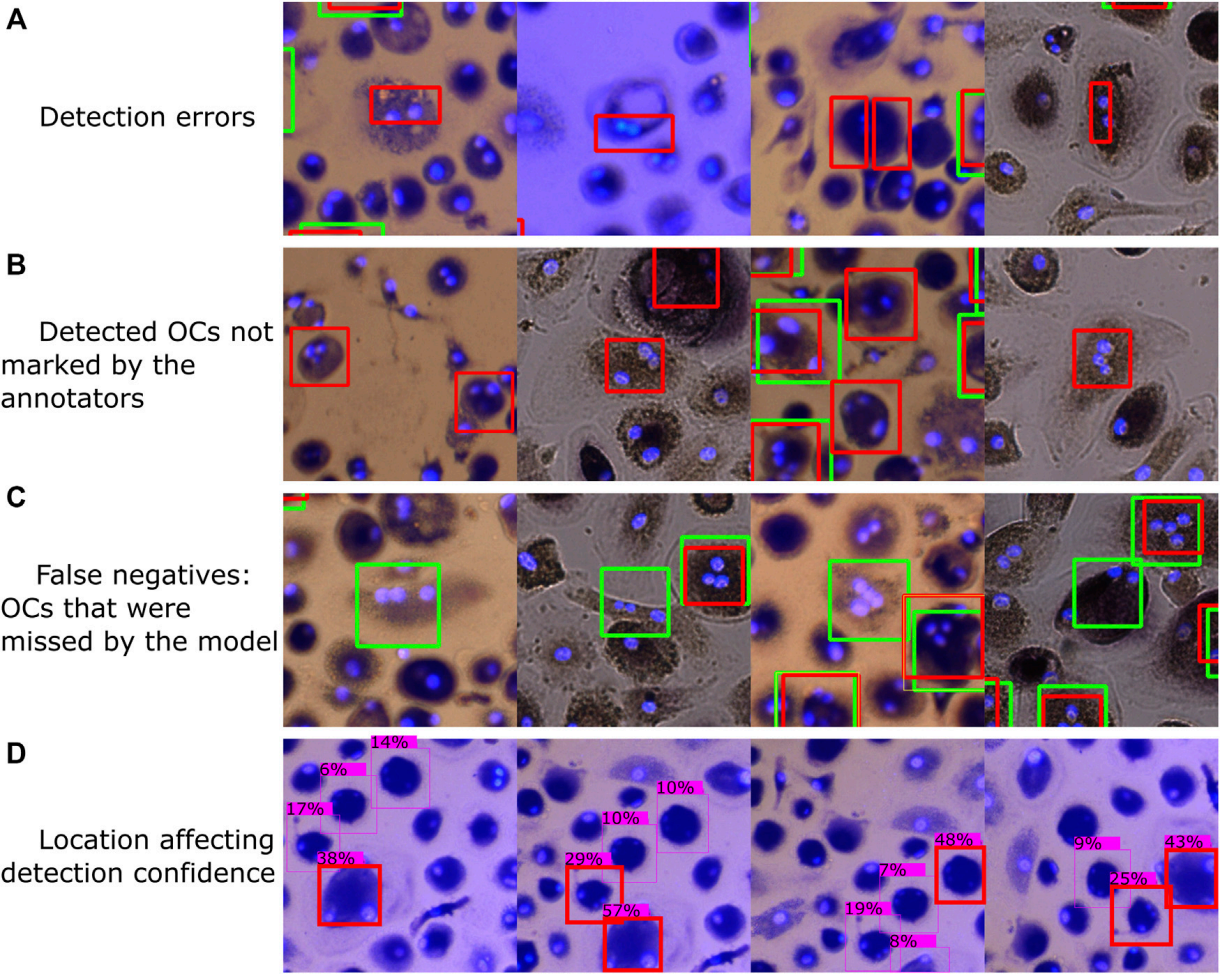
Typical errors, including **(A)** classifying parts of a cell as an OC, **(B)** OCs regarded as false positives during evaluation due to the annotators missing some OCs during manual counting, **(C)** OCs differing in shape and appearance from most OCs in our dataset that were found by the annotators but not by the model, and **(D)** cell location affecting detection confidence. Red bounding boxes represent detections made by model M1, with green representing the annotated labels (ground truth).

A few samples of typical errors are shown in Figure 11. Since the object detection models can only process small images, thus having to divide each well image into smaller segments, some of the training images will contain incomplete OCs that remain marked. The model has thus learned to recognize such incomplete OCs, resulting in detection errors, as shown in Figure 11A. A segmentation algorithm that considers the labeled OCs so that none of the segments contain incomplete OCs could be developed, which would remove such erroneous samples from the training data.

Figures 11B,C shows some false positives and false negatives with respect to the human-marked OCs. After going through some of the automatically analyzed wells, we found several false positive detections by the trained model that should be considered true positives, resulting from the annotators not detecting each OC during manual counting. Although this is not a limitation of the trained model, it shows that human error can affect the evaluation of the model. To further improve the detection accuracy, a manual evaluation of the model’s and human’s detections can be performed to correct erroneous and missing labels in the training data and then retrain the model. Many false negatives, i.e., human-labeled OCs that the model did not detect, had different shapes and appearances than most of the OCs in our dataset. In addition, a few faulty labels were caused by accidental clicks using the Fiji marking tool.

A small translation was applied to the first three image segments shown in Figure 11D, with the fourth segment being rotated, which resulted in different detection confidences for the same cells. Since a detection threshold is required to filter out erroneous detections and improve accuracy, the well images’ segmentation approach can affect the results. For example, the method of detecting OCs using overlapping image segments, as shown in Figure 2 and Figure 3B, resulted in more detections (a significant, 12.5% bias) compared to using non-overlapping segments (2.1% bias). Therefore, it is important to validate the model and segmentation approach to tune the detection threshold.

It is required that the cells are approximately the same size relative to the image segments when using our trained model. Darknet models can be trained with image sizes between 320 and 608 pixels in width and height, where we used 416 pixels. Using images with different sizes is also possible, as demonstrated in scenario four, which will be resized accordingly when processed through Darknet. Therefore, the image segmentation strategy must be adjusted based on cell size and the size of the whole well image. If detection of larger cells or the number of nuclei per cell is required, our model can be retrained with new labeled data to increase accuracy. The approach can also be used on completely different cell types by training new models and can be used in real-time systems with a prediction time of roughly 26 images per second, depending on hardware.

## 5 Conclusion

An approach for automatically detecting and counting osteoclasts in microscopic images has been developed and evaluated. Several object detection (deep learning) models were trained, validated, and tested using 307 different wells from seven human PBMC donors, containing a total of 94,974 marked OCs. The first model was optimized for the OC counting task by utilizing a train, validation, and test split on all the available data, resulting in a test accuracy of 85.26% mAP@0.1. The model counted on average 2.1% more OCs per well than the human annotators, with limits of agreement between 23.8% and -19.6%, an RMSE of 37.29, and a correlation coefficient of 0.99. The approach can generalize across different experiments with effective data augmentation, supporting the potential adaptation of the model in different studies. Furthermore, two independent annotators agreed less than the trained models on the same dataset. A substantial amount of labor and time can thus be saved by automatically detecting OCs with (at least) human-level accuracy and reliability while reducing operator bias. Additionally, the trained model can be continuously improved by introducing new data from different experiments.

## Data Availability

The raw data supporting the conclusions of this article will be made available by the authors, without undue reservation.
